# Targeting FGF21 in cardiovascular and metabolic diseases: from mechanism to medicine

**DOI:** 10.7150/ijbs.73936

**Published:** 2023-01-01

**Authors:** Huiling Tan, Tong Yue, Zhengfang Chen, Weiming Wu, Suowen Xu, Jianping Weng

**Affiliations:** 1 Department of Endocrinology, Institute of Endocrine and Metabolic Diseases, The First Affiliated Hospital of USTC, Division of Life Sciences and Medicine, Clinical Research Hospital of Chinese Academy of Sciences (Hefei), University of Science and Technology of China, Hefei, Anhui, 230001, China.; 2 Changshu Hospital Affiliated to Soochow University, Changshu No.1 People's Hospital, Changshu 215500, Jiangsu Province, China.; 3 Changshu Hospital Affiliated to Nanjing University of Chinese Medicine, Changshu, China.

**Keywords:** fibroblast growth factor 21, cardiovascular diseases, metabolic diseases, targets, therapeutics

## Abstract

Cardiovascular and metabolic disease (CVMD) is becoming increasingly prevalent in developed and developing countries with high morbidity and mortality. In recent years, fibroblast growth factor 21 (FGF21) has attracted intensive research interest due to its purported role as a potential biomarker and critical player in CVMDs, including atherosclerosis, coronary artery disease, myocardial infarction, hypoxia/reoxygenation injury, heart failure, type 2 diabetes, obesity, and nonalcoholic steatohepatitis. This review summarizes the recent developments in investigating the role of FGF21 in CVMDs and explores the mechanism whereby FGF21 regulates the development of CVMDs. Novel molecular targets and related pathways of FGF21 (adenosine 5'-monophosphate-activated protein kinase, silent information regulator 1, autophagy-related molecules, and gut microbiota-related molecules) are highlighted in this review. Considering the poor pharmacokinetics and biophysical properties of native FGF21, the development of new generations of FGF21-based drugs has tremendous therapeutic potential. Related preclinical and clinical studies are also summarized in this review to foster clinical translation. Thus, our review provides a timely and insightful overview of the physiology, biomarker potential, molecular targets, and therapeutic potential of FGF21 in CVMDs.

## 1. Introduction

**Figure 1:**
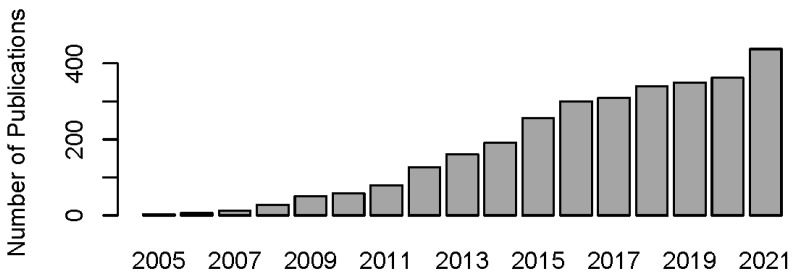
** Year publications of FGF21 in PubMed (date of search, March 1, 2022).** The number of publications on FGF21 is increasing year by year.

**Figure 2:**
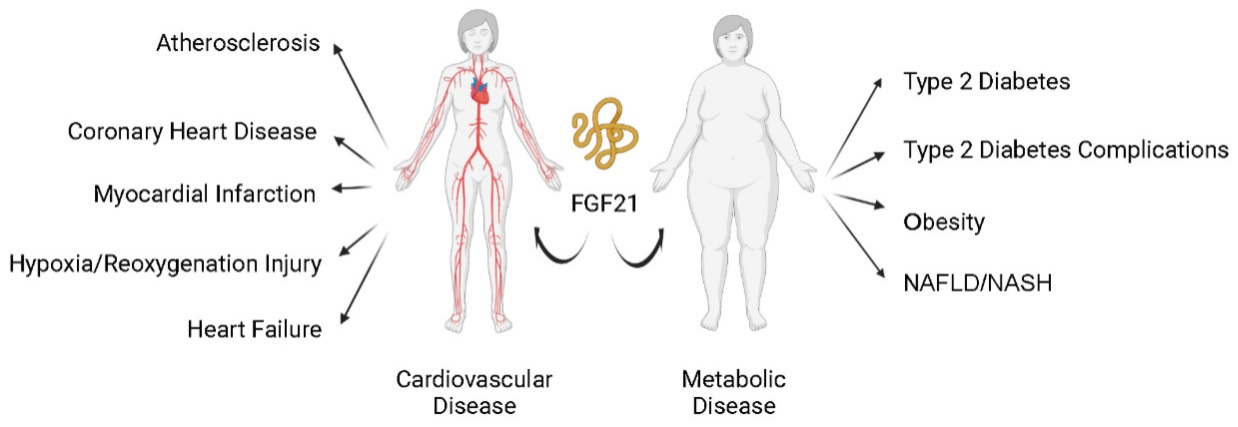
** FGF21 and cardiovascular and metabolic diseases.** Current studies support that FGF21 plays an important role in cardiovascular and metabolic diseases. Nonalcoholic fatty liver disease (NAFLD); nonalcoholic steatohepatitis (NASH).

Modern human society is burdened with the pandemic of cardiovascular diseases (CVD, such as atherosclerosis, myocardial infarction (MI), and heart failure (HF)) and metabolic diseases (such as diabetes mellitus (DM), obesity, and metabolic syndrome (MS)). Cardiovascular and metabolic disease (CVMD) is the leading cause of mortality and morbidity worldwide and is mostly caused by abnormal metabolic processes 1, 2. Fibroblast growth factor 21 (FGF21), which has protective effects against damage induced by abnormal metabolic conditions, has been intensively studied (Figure 1) and widely identified as a therapeutic candidate for CVMDs (Figure 2) 3, 4.

FGF21, a signaling protein of 208 amino acids, belongs to the fibroblast growth factor (FGF) family 5. The human FGF family includes 22 members and has been divided into seven subfamilies. Subfamily 19 comprises three endocrine factors: FGF21, FGF19, and FGF23. The rest of the other subfamilies work in an intracrine or paracrine manner. Unlike conventional functions of promoting fibroblast proliferation and growth mediated by other FGFs, subfamily 19 mainly focuses on regulating metabolism. FGF19 regulates bile acid homeostasis, FGF23 regulates phosphate homeostasis, and FGF21 attracts the most attention due to its multiple beneficial effects on regulating glucose and lipid homeostasis as well as energy metabolism 6. The way that transmembrane tyrosine kinase FGF receptor (FGFR) signaling activity is activated between subfamily 19 and other subfamilies is also different. Nonendocrine FGFs require heparan sulfate as a coreceptor to activate FGFR. However, endocrine FGFs lack the canonical heparin-binding domain (which also permits them to escape into the blood and be similar to endocrine hormones). They form a cell surface receptor complex with FGFR and another coreceptor, β-Klotho (KLB) 7.

## 2. FGF21: tissue expression and biological functions

**Figure 3:**
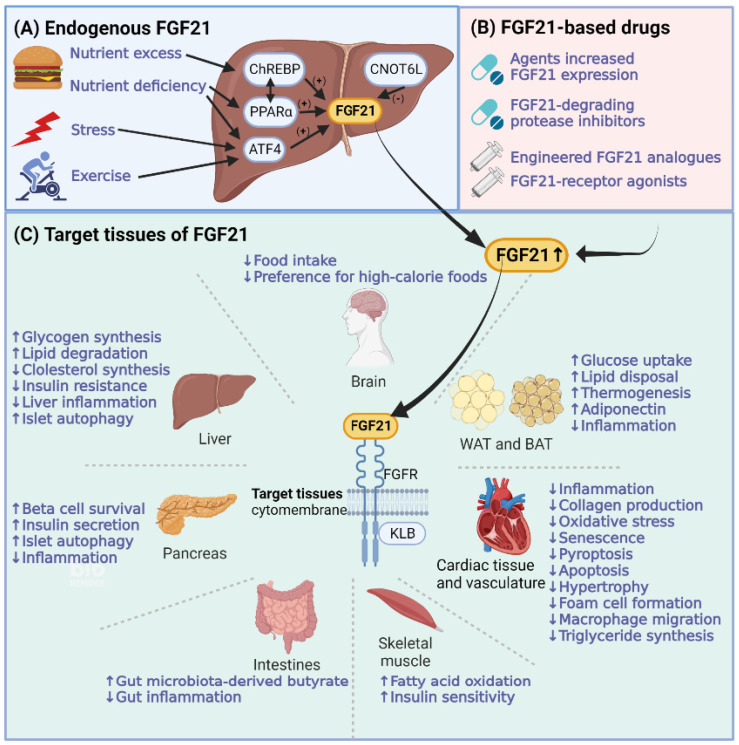
** Molecular functions of FGF21 in cardiovascular and metabolic diseases. (A)** The process of hepatic FGF21 production with different activating transcription factors, including peroxisome proliferator-activated receptors (PPARs), activating transcription factor 4 (ATF4), carbohydrate response element-binding protein (ChREBP), and CCR4-NOT transcription complex subunit 6-like (CNOT6 L). **(B)** FGF21-based drugs that can increase FGF21 levels. **(C)** FGF21 forms a cell surface receptor complex with FGFR and another coreceptor, β-Klotho (KLB). FGF21 exerts its metabolic protective effects by coordinating target tissues of FGF21, including the brain, liver, adipose tissues, pancreas, cardiac tissue and vasculature, muscles, and intestines. The brain can regulate food preferences and assist in coordinating FGF21-mediated interorgan responses. The liver and adipose tissues are the main targets of FGF21. The direct action of hepatic FGF21 is to regulate lipid and free fatty acid metabolism in the liver. The actions of FGF21 in white adipose tissue (WAT) and brown adipose tissue (BAT) are mainly focused on lipolysis, increasing glucose uptake, and thermogenesis.

FGF21 is expressed after being induced by various stress conditions, such as cold exposure, nutritional stresses, exercise, and some pathologic conditions 8-11. Tissues expressing FGF21 include the liver, brown adipose tissue (BAT), white adipose tissue (WAT), muscle, pancreas, heart, and brain 5, 12. The liver is the major source of serum FGF21 under physiological conditions 13. The process of hepatic FGF21 production is presented in Figure 3A. Different FGF21 transcription factors (including peroxisome proliferator-activated receptor (PPAR) α, activating transcription factor 4 (ATF4), carbohydrate response element-binding protein (ChREBP), and CCR4-NOT transcription complex subunit 6-like (CNOT6 L)) can be regulated under different states to promote FGF21 expression. Hepatic FGF21 can be induced in both states of nutrient deficiency (starvation, ketogenic diet, and methionine/choline-deficient diet) and forms of nutrient excess (simple sugars). Under nutrient-deficient states, fatty acids activate the transcription factor PPAR α, whose downstream target is FGF21 8. ATF4 is another effector that induces FGF21 expression under nutrient deficiency (amino acid limitation) 14 and oxidative stress 15, 16. However, under conditions of carbohydrate load, in the liver and adipose tissues, another factor named ChREBP induces FGF21 to regulate *de novo* lipogenesis 17. Notably, hepatic PPARα is required for the carbohydrate load-induced induction of FGF21 by ChREBP 18. Moreover, CNOT6L is the catalytic subunit of the CCR4-NOT deadenylase, which can promote FGF21 mRNA decay. Disruption of CNOT6L activity in the liver can increase serum FGF21 levels 19.

FGF21-based drugs have also been investigated to increase FGF21 levels, which are summarized in Table 1 and Figure 3B. FGF21-based drugs can be divided into four categories: agents that increase FGF21 expression, FGF21-degrading protease inhibitors, FGF21 analogs, and FGF21-receptor agonists. For agents that increase FGF21 expression, in addition to chemicals such as neohesperidin 20 that increase FGF21 expression directly, the expression of FGF21 is increased by FGF21-transcription factor modulators, such as PPARα agonists (Pemafibrate) 21, cAMP-responsive element-binding protein H modulator (MS-275) 22, ATF4 modulator (Carbon monoxide) 23, and CNOT6L inhibitor (iD1) 24. In addition, agents used for DM treatment, such as renal sodium/glucose cotransporter-2 inhibitors (Canagliflozin) 25, glucagon-like peptide-1 (GLP-1) analog (Liraglutide) 26, and metformin 27, also increase the expression of FGF21. Regarding FGF21-degrading protease inhibitors, fibroblast activation protein inhibitors can increase endogenous FGF21 levels in cynomolgus monkeys 28. FGF21 analogs and FGF21-receptor agonists are the most studied FGF21-based drugs in preclinical and clinical studies. The preclinical and clinical studies of FGF21-based drugs in CVMDs will be discussed later.

Target tissues of FGF21 comprise the liver, adipose tissues, brain, pancreas, heart, and kidney. FGF21 mediates biological functions in target tissues with the assistance of KLB and FGFR 29. The molecular functions of FGF21 in CVMDs are summarized in Figure 3C. As a hepatoprotective factor, the direct action of hepatic FGF21 is to regulate lipid and free fatty acid metabolism in the liver, protecting against steatosis caused by nutrient stressors 30. Adipose tissue is another essential target of hepatic FGF21. The endocrine actions of FGF21 in adipocytes are mainly focused on lipolysis and increasing glucose uptake. Weight loss caused by increased fatty acid oxidation was found to be as expected. However, the level of blood glucose is not decreased as expected in humans, which indicates the possibility of FGF21 as a lipid regulator analogous to insulin as a glucose regulator under the underlying different glucose control modes between animals and humans 31. In addition, hepatic FGF21 can penetrate the blood-brain barrier (BBB). FGF21 derived from the liver can act on neurons, decrease sweet and alcohol preferences and stimulate water intake to maintain balanced nutritional conditions 32. Moreover, FGF21 has nonmetabolic effects on daily wheel-running behavior as well as infertility and growth, which is associated with FGF21 signal transduction in the hypothalamus and brainstem 33. Notably, muscle is another important source of serum FGF21. Myocytic FGF21 can act on WAT to induce the browning of WAT 34, on skeletal muscle to protect against insulin resistance 35, and on the heart to protect against cardiac hypertrophy 36.

Beyond its endocrine actions, FGF21 acts in an autocrine/paracrine way in BAT, WAT, the heart, and the pancreas. In adipose tissue, autocrine-derived FGF21 can induce cold-induced thermogenesis by uncoupling protein 1 (Ucp1)-dependent or Ucp1-independent mechanisms 37. Cold exposure-induced FGF21 in BAT and WAT also promotes adipocyte browning, which can increase thermogenesis, with the assistance of coactivator peroxisome proliferator-activated receptor-gamma coactivator-1alpha and cytokine C-C motif chemokine ligand 11 38. In the pancreas, the secretion of FGF21 and digestive enzymes in pancreatic acinar cells is synchronized, which can minimize proteotoxic stress and prevent protein overload. Increased FGF21 also helps the pancreas protect against injury caused by pancreatitis in mice, such as endoplasmic reticulum stress and inflammation 39. FGF21 can protect β cells from stress-induced dysfunction and induce insulin gene expression 40. In addition, the autocrine action of FGF21 in the heart is stimulated by cardiomyopathy. In this way, FGF21 protects against heart disease injuries such as cardiac hypertrophy and infarction 36.

## 3. FGF21 and CVD

### 3.1 Atherosclerosis and coronary artery disease (CAD)

#### 3.1.1 Relationship between FGF21 and CAD

CAD is a critical form of CVD. The elevated level of FGF21 is a biomarker for higher CVD risk in statin-treated patients 41. In DM patients with coronary artery calcification, a lower level of FGF21 predicts a better long-term prognosis 42. One study indicated that the baseline level of serum FGF21 can predict incident CAD in people with type 2 diabetes mellitus (T2DM) without known CVD 43. However, another multiethnic study did not support FGF21 as a CVD biomarker in atherosclerosis patients without known CVD 44. Although population characteristics for FGF21 as an incident CVD biomarker are controversial, FGF21 levels may still be utilized as a CAD risk biomarker for primary prevention 43. The CAD primary prevention effect of FGF21 has been represented in subclinical atherosclerosis patients without nonalcoholic fatty liver disease (NAFLD) 45 and patients without a history of DM 46. Moreover, FGF21 can be a biomarker to predict subclinical atherosclerosis, the pathological basis of CAD, in women without a history of CVD 47.

#### 3.1.2 Mechanisms of FGF21 against atherosclerosis

The elevated FGF21 plays a protective role in CAD. Atherosclerosis is an important pathological basis of CAD. FGF21 can protect against atherosclerosis by modulating various proatherogenic cellular events. First, FGF21 can alleviate endothelial dysfunction via multiple pathways. For example, coculture of human umbilical vascular endothelial cells (HUVECs) with 5 ng/mL FGF21 delayed endothelial senescence by increasing silent information regulator 1 (SIRT1) 48. One *in vitro* study investigated the impact of FGF21 on HUVEC pyroptosis stimulated by oxidized low-density lipoprotein, and the authors found that FGF21 can inhibit HUVEC pyroptosis through the tet methylcytosine dioxygenase 2- ubiquinol cytochrome c reductase core protein I-reactive oxygen species pathway 49. In apolipoprotein E^-/-^ (apoE^-/-^) mice, FGF21 inhibits the Fas signaling pathway to decrease endothelial apoptosis 50, and the effects on endothelial pyroptosis were mediated by inhibition of the NOD-, LRR- and pyrin domain-containing 3 (NLRP3) inflammasome pathway 51. In addition, one *in vivo* study reported that FGF21 inhibits the nuclear factor kappa light chain enhancer of the activated B-cell (NF-κappaB) pathway in endothelial cells to improve atherosclerosis 52.

Second, macrophages are also crucial to the development of atherosclerosis. *In vitro* experiments proved that FGF21 can inhibit macrophage-derived foam cell formation to improve atherosclerosis 53. FGF21-induced autophagy can reduce cholesterol accumulation and increase cholesterol efflux from foam cells via the activated kinase C receptor 1-adenosine 5'monophosphate-activated protein kinase (AMPK) pathway *in vivo* 54. In an *in vitro* study, researchers also found that the cholesterol efflux-promoting effects of FGF21 are mediated by inducting ATP binding cassettes A1 and G1 in foam cells 55. Moreover, FGF21 can affect macrophage-related cholesterol efflux by regulating the microRNA-33-sterol regulatory element-binding protein pathway 56. Apart from inhibiting foam cell formation, FGF21 also suppresses macrophage apoptosis to improve atherosclerosis *in vitro* 57.

Third, neointimal hyperplasia, involving the proliferation and migration of vascular smooth muscle cells, is another essential pathological step for atherosclerosis. In high-fat diet (HFD) mice treated with a low dose of streptozotocin, FGF21 inhibits the migration and proliferation of vascular smooth muscle cells through the FGFR1-spleen tyrosine kinase-NLRP3 inflammasome pathway 58. Notably, FGF21 can promote the secretion of adiponectin, which also exerts an atheroprotective effect by reducing endothelial dysfunction, thereby blocking the conversion of macrophages to foam cells and inhibiting vascular smooth muscle cell proliferation 59.

In conclusion, FGF21 can modulate proatherogenic cellular events, including vascular endothelial cells, macrophages, and vascular smooth muscle cells, to protect against atherosclerosis and CAD.

#### 3.1.3 Preclinical and clinical trials of FGF21 in atherosclerosis

The protective effect of FGF21 against atherosclerosis has been proven in many preclinical trials. One study carried out on apoE^-/-^ mice indicated that the administration of recombinant human FGF21 (rhFGF21) can protect against atherosclerotic plaque formation 60. In another study, apoE^-/-^ mice were treated with an FGF21 analog (LY2405319). LY2405319 treatment also reduces atheromatous plaque and lowers lipid profiles 61 in hypercholesterolaemic apoE*3-Leiden. In CETP mice (a model that has a lipid profile similar to that of human patients), rhFGF21 administration can reduce the severity and increase the stability index of atherosclerotic lesions 62. However, there have been no clinical trials about the application of FGF21-based drugs in CVD treatment, and most clinical trials have investigated the application of FGF21 in metabolic diseases.

### 3.2 Myocardial infarction (MI) and ischemia/reperfusion (I/R) injury

#### 3.2.1 Relationship between FGF21 and MI

MI is a severe ischemic heart disease with high mortality caused by coronary atherosclerosis and CAD. One study involving acute MI patients and stable angina pectoris (AP) patients showed that MI patients have higher serum FGF21 levels than stable AP patients 63. Another study indicated that FGF21 has precise predictability for increased risks of unstable AP 64. One study that enrolled 183 acute MI patients and 55 patients without acute MI suggested that circulating FGF21 levels might be a predictive marker for the clinical outcomes of acute MI patients 65. Another study enrolled 348 MI patients with ST-segment elevation and found that elevated FGF21 is a powerful predictor of major adverse cardiovascular events 66.

#### 3.2.2 Mechanisms of FGF21 against MI

FGF21 can regulate cardiac myocyte, H9c2 cell, and fibroblast actions to improve MI. In cardiac myocytes, FGF21 protects myocytes against ischemic injury 63. In addition, FGF21 can effectively suppress inflammation and fibrosis in myocytes by regulating the FGFR-early growth response protein 1 pathway 67. In H9c2 cells, apoptosis and oxidative stress can be alleviated via the FGF21-phosphatidylinositol 3-kinase (PI3K)-protein kinase B (Akt) pathway 68. Regarding cardiac fibroblasts, FGFF21 can promote fibroblast apoptosis and reduce collagen production by inactivating the transforming growth factor-β1-drosophila mothers against the decapentaplegic protein 2/3-matrix metalloproteinase 2/9 signaling pathway in mice with myocardial infarction 69.

On the other hand, FGF21 can effectively suppress ventricular arrhythmia post MI by regulating the microRNA-143-early growth response protein 1 axis *in vivo* 70. Another study confirmed the influence of FGF21 on decreasing the incidence of arrhythmia in rat and guinea pig models and showed that the incidence of ischemic arrhythmias in rhFGF21-treated models was decreased. Consistently, in hydrogen peroxide-treated AC16 cells, administration of FGF21 shortens cell action potential duration by ameliorating voltage-gated sodium channel 1.5 and potassium inwardly rectifying channel 2.1 dysregulations 71.

In conclusion, FGF21 can improve MI and ventricular arrhythmia post MI by regulating the inflammation, fibrosis, and action potential duration of myocytes.

#### 3.2.3 Mechanisms of FGF21 against I/R injury

The rapid restoration of blood perfusion after MI may lead to I/R injury, which aggravates cell injury and death in the ischemic heart. FGF21 exhibits protective effects against heart I/R injury, and the mechanism has been investigated in animal and cell models. In the rat myocardial I/R model, researchers injected FGF21 into the rat myocardial I/R model and found that FGF21 can decrease infarction and inflammation after I/R 72. In the H9c2 hypoxia/reoxygenation model, FGF21 can improve H9c2 cell I/R injury by promoting autophagy via different pathways, such as the FGF21-Beclin‑1/vacuolar protein sorting 34 proteins-autophagy pathway 73 and the FGF21-microRNA-145- autophagy pathway 72. FGF21 can also decrease the expression levels of galectin-3 to alleviate hypoxia-induced cardiac myocyte injury *in vivo* 74. In addition, FGF21 can improve the energy metabolism of H9c2 cells after I/R via the FGF21-angiopoietin-2-glucose transporter 1 pathway *in vitro* 75.

#### 3.2.4 Preclinical and clinical trials of FGF21 in MI

In an animal model, intramuscular injection of FGF21-expressing adenoviral vectors into MI mice improved left ventricular systolic dysfunction after two weeks with the assistance of adiponectin 76. In FGF21 knockout MI mice, FGF21 deficiency weakens the aerobic exercise-induced inhibition of oxidative stress and apoptosis 68. Moreover, exercise training increased FGF21 expression and alleviated oxidative stress and cardiac fibrosis after MI 68, 69. However, clinical trials about the application of FGF21 in CVD patients are still lacking and remain to be investigated.

### 3.3 Cardiac remodeling and HF

#### 3.3.1 Relationship between FGF21 and HF

FGF21 has predictive value for the development and progression of HF. One study obtained blood and tissue samples from patients with end-stage HF with reduced ejection fraction (HFrEF) and found elevated serum FGF21 in HFrEF patients 77. Another study that enrolled 128 HFrEF patients and 71 controls also found elevated FGF21 levels in patients with HFrEF 78. In addition to HFrEF, patients with HF with preserved ejection fraction (HFpEF) also have higher FGF21 levels than patients without HF, which shows that FGF21 has the potential to diagnose HFpEF 79 and reflect diastolic dysfunction 80.

#### 3.3.2 Mechanisms of FGF21 against HF

FGF21 can improve HF by regulating cardiac remodeling, which has been investigated in animal and cell models. Cardiac remodeling is a pathological process associated with cardiac hypertrophy, fibrosis, and other harmful cardiac derangement changes, which eventually cause HF. The protective effects of FGF21 on preventing cardiac hypertrophy have been evaluated in pathological animal models such as uremic cardiomyopathy rats 81, obesity-associated cardiomyopathy mice 82, and hypertension mice 51. All of these pathological cardiomyopathies can result in HF. Moreover, in angiotensin II-induced cardiac hypertrophy mice, FGF21 can regulate the deacetylase activity of SIRT1 to improve cardiac hypertrophy 83. For *in vitro* studies, FGF21 treatment can protect cardiomyocytes from hypertrophy and inflammation against oxidative stress 84, the possible factor that leads to HF 85. Apart from improving cardiac hypertrophy, FGF21 can attenuate cardiac derangements to inhibit cardiac remodeling and HF. In FGF21^-/-^ mice fed an HFD, the impaired ability of FGF21 promoted autophagy in cardiomyocytes, leading to lipid accumulation and cardiac derangements 82. Pathological cardiac fibrosis is another critical step toward HF, and the protective effects of FGF21 against fibrosis are also observed in hypertensive heart disease mouse models 86.

#### 3.3.3 Preclinical and clinical trials of FGF21 in HF

Preclinical and clinical trials of FGF21 in HF are limited. One study delivered 3 longevity-associated genes, FGF21, into HF mouse models and found increased heart function in aortic constriction 87. Moreover, DPP-4 inhibitor-induced FGF21 also improves the contractile efficiency of pressure-overloaded mouse hearts 88.

## 4. FGF21 and metabolic diseases

### 4.1 T2DM

FGF21 can be a biomarker for predicting the onset of DM and has therapeutic effects on T2DM and its complications. Mechanisms of FGF21 against T2DM include improving glucose homeostasis, attenuating obesity, and alleviating inflammation. FGF21-based drugs have also been designed to treat T2DM in many preclinical and clinical studies.

#### 4.1.1 Relationship between FGF21 and T2DM

A recent meta-analysis of 317 articles revealed that T2DM patients have a higher serum FGF21 level than control groups 89. Moreover, serum FGF21 levels are lower in T2DM patients with higher urinary glucose excretion levels 90. Another study assessed the association between FGF21 and glycohemoglobin, beta-cell function, and insulin resistance among 2 584 patients without hypoglycemic agent treatment. They found that patients with a higher FGF21 level have a higher risk of glycemic progression over five years 91. The FGF21/adiponectin ratio is another valuable biomarker to predict the deterioration in glycemia 92. Moreover, one study observed the DM incidence of 1 380 nondiabetic subjects over nine years and measured serum levels of FGF21. The results showed that serum FGF21 appears to be an alternative to the glucose tolerance test as a biomarker for DM prediction 93. All of those studies 91-93 showed that FGF21 might be a biomarker in predicting the onset of prediabetes 92 and DM 93. FGF21 is also related to the transition from prediabetes to DM. A recent study found that in WNIN/GR-Ob rats, the attenuation of FGF21 may aggravate glucose impairment and the transition from prediabetes to DM 94.

#### 4.1.2 Mechanisms of FGF21 against T2DM

Elevated FGF21 plays a protective role in T2DM, which has been observed in animal models. In New Zealand obese mice, a T2DM and obesity model, data suggested that dietary methionine restriction-induced FGF21 can protect mice from glucose intolerance and T2DM 95. Another study demonstrated that alternating-day fasting can inhibit insulin resistance and obesity in leptin receptor knockout mice, possibly depending on FGF21 96.

The mechanism by which FGF21 protects against T2DM mainly involves improving glucose homeostasis in various ways. First, FGF21 can promote insulin expression and secretion to protect against T2DM. In that study, researchers found that FGF21 knockout exacerbates islet beta-cell failure and suppresses insulin secretion in pancreatic islets of *db/db* mice. Then, they found that inhibition of PI3K/Akt signaling can effectively suppress FGF21-induced insulin expression in mice, which indicated that FGF21-induced insulin expression and secretion occurred through the PI3K/Akt signaling pathway 97. Notably, clamp studies in adults with T2D revealed that the secretion of insulin instead of glucose consumption induces a postprandial FGF21 increase 98, which indicated that FGF21 is also an insulin-dependent hormone in humans.

Second, FGF21 can improve glucose homeostasis by protecting β cells. FGF21 can downregulate islet cell lipid accumulation and thereby reduce the apoptosis and dysfunction of β-cells *in vivo* and *in vitro*, which is probably via activation of the AMPK-acetyl coenzyme A carboxylase (ACC) and PPARδ/γ signaling pathways 99. Autophagy serves another essential role in protecting β cell survival and function. FGF21 can induce islet autophagy by inhibiting the AMPK-mammalian target of rapamycin (mTOR) signaling pathway in HFD C57/BL6J mice 100. Apart from insulin-dependent ways to improve glucose homeostasis, FGF21 can take up glucose in adipocytes in an insulin-independent manner. Pegylated FGF21 can normalize plasma glucose in streptozotocin-treated mice and competitive insulin receptor antagonist-treated mice, whereas the function of pancreatic β-cells does not rest 101.

Third, FGF21 can reduce serum insulin and increase insulin sensitivity to protect against systemic insulin resistance. One study observed early-life exposure to chronic stress-induced long-term insulin sensitivity along with substantially increased FGF21 levels in C57Bl/6J mice 102. To determine the specific target of FGF21 in improving hepatic insulin sensitivity, researchers administered FGF21 in mice and found that FGF21 can increase insulin sensitivity by inhibiting liver mTORC1. Consistently, mTORC1 is activated, and hepatic insulin resistance is exacerbated in FGF21-deficient mice 103. Moreover, subcutaneous fat tissue is an essential tissue that FGF21 targets to regulate systemic insulin sensitivity. FGF21 can promote the expansion of subcutaneous fat and elevate adiponectin levels in subcutaneous fat to protect against systemic insulin resistance *in vivo* 104. Adiponectin is an essential protein that can help FGF21 mediate insulin sensitivity. Researchers administered FGF21 to tunicamycin-induced 3T3-L1 adipocytes and HFD-induced obese mice. They found that the administration of FGF21 can inhibit endoplasmic reticulum stress in adipose tissue to release the suppression of adiponectin expression and restore insulin signaling 105.

In addition to improving glucose homeostasis, FGF21 can protect against T2DM in other ways. On the one hand, FGF21 can increase energy expenditure and attenuate obesity, as shown in obese mice 106. However, the function of FGF21 in preventing diet-induced T2DM is not always accompanied by body fat mass change. In New Zealand obese mice, FGF21 treatment increases energy expenditure by WAT browning without changing body fat mass 107. On the other hand, FGF-21 can alleviate inflammation while improving hyperglycemia. One study's results demonstrated the potent anti-inflammatory effect of FGF21 in the serum and WAT in *db/db* mice. *In vitro* experiments showed that FGF21 can ameliorate glucose uptake of tumor necrosis factor-α-induced insulin resistance by inhibiting the NF-κappaB signaling pathway in 3T3-L1 adipocytes 108.

#### 4.1.3 Effects of FGF21 on complications of T2DM

FGF21 has therapeutic effects on complications of T2DM, including diabetic macroangiopathies and microangiopathies.

The heart is a vital target organ for T2DM, and the role of FGF21 in T2DM cardiovascular complications has been investigated in studies. Evidence suggests that FGF21 has therapeutic effects on diabetic cardiomyopathy *in vitro* and *in vivo* 109. In T2D mice, FGF21 can prevent T2DM lipotoxicity-induced cardiomyopathy by activating both the AMPK-ACC-carnitine palmitoyltransferase-1-mediated lipid-lowering pathway and the AMPK-Akt2-nuclear transcription factor-E2-related factor 2 (Nrf2)-mediated antioxidative pathway in the heart 110. Moreover, in T2DM patients with HFpEF, FGF21 showed its diagnostic potential for HFpEF 111. Another study stratified 1132 DM patients with coronary artery calcification based on their FGF21 levels and recorded their major adverse cardiovascular events during 1.5-5.1 years of follow-up. Study results concluded that a lower baseline level of FGF21 predicts a better cardiovascular prognosis in DM patients 112. Another study also supported that the level of circulating FGF21 is positively associated with human aortic stiffness, which was investigated in 130 T2DM patients 113. *In vitro*, FGF21 can direct or activate the calcium/calmodulin-dependent protein kinase 2-AMPKα signaling pathway to attenuate high glucose-induced aortic endothelial function impairment and enhance aortic endothelium-dependent vasorelaxation 114.

Cerebrovascular disease is another severe complication of T2DM. The ischemic stroke mortality of T2DM patients is higher and neurological outcomes are worse than ordinary people 115. Daily rhFGF21 administration lasting for two weeks post stroke in T2DM *db/db* mice can improve white matter and cerebrovascular impairment 116. One study showed that FGF21 improves neurological outcomes after ischemic stroke in T2DM *db/db* mice, which may contribute to the antiproinflammatory effects by activating PPARγ in the brain 117. In addition to cerebrovascular disease, DM tends to induce BBB dysfunction and cognitive impairment, including Alzheimer's disease 118. In T2DM mice, massive BBB leakage was observed after ischemic stroke. FGF21 can protect against BBB disruption by activating PPARγ after ischemic stroke in T2DM *db/db* mice 119. A study in T2DM mice demonstrated another potential mechanism of decreasing BBB permeability via the FGFR1-Kelch ECH-associated protein 1-Nrf2 activation pathway 120.

In addition to diabetic macroangiopathy, FGF21 also plays a role in diabetic microangiopathies such as diabetic retinopathy and diabetic nephropathy. One cross-sectional study recruited a total of 654 T2DM patients and found that the risk of sight-threatening diabetic retinopathy increased as the serum FGF21 level exceeded 554.69 pg/mL 121. In type 1 diabetic mice, FGF21 can prevent early diabetic retinopathy by protecting photoreceptor function 122.

Regarding the association between FGF21 and diabetic nephropathy, a 6.3-year prospective cohort study in women with T2DM indicated that FGF21 can independently predict the progression to end-stage renal disease 123. Another study found that the combination of FGF21 and soluble tumor necrosis factor receptor type 1 can better predict renal outcomes in a 3.5-year follow-up period for T2DM patients 124. Moreover, from the aspect of genetic variants, study results indicated that FGF21-associated genetic variations are related to the estimated glomerular filtration rate in DM patients 125.

#### 4.1.4 Preclinical and clinical trials of FGF21 in T2DM

Many FGF21-based drugs have been designed to treat T2DM, such as pegbelfermin (BMS-986036), a PEGylated FGF21. The results from a randomized phase 2 study in T2DM patients with obesity showed that pegbelfermin compared with placebo can improve triglycerides and increase adiponectin levels. However, there were no significant differences observed in glycated hemoglobin glycosylated hemoglobin A1c. The adverse effects of pegbelfermin are mild and mainly focused on injection-site bruising and diarrhea 126. To overcome the weak binding affinity and instability of natural FGF21 127, researchers analysed the structural and dynamic information of FGF21 and developed many FGF chimeras. For example, researchers devised a paracrine-endocrine FGF chimera that substituted the core of FGF21 with a stable and high receptor affinity core from paracrine FGF1 128. Another study using nuclear magnetic resonance spectroscopy found that the noncanonical flexible β-trefoil conformation of FGF21 affects protein stability, and they designed an FGF21-FGF19 chimera that can better decrease blood glucose in *ob/ob* mice 129. FGF21 gene therapy is another way to overcome the unfavorable pharmacokinetic properties of native FGF21. Researchers administered an FGF21-overexpressing adeno-associated viral vector to mouse visceral adipose tissue, which decreases body weight and improves insulin sensitivity 130. This study also highlights the potential of gene therapy to overexpress FGF21 in target tissues. Another study utilized lentivirus to mediate human FGF21 stable expression in T2DM rat liver tissue. The results showed that genes related to insulin sensitivity are increased, suggesting that injecting lentivirus-mediated rhFGF21 into rat livers might effectively treat T2DM 131.

A previous clinical study indicated that the lipid-lowering effect of FGF21 is more significant than that of hypoglycemia in humans 126, enlightening us to explore the potential of applying FGF21 with clinical antidiabetic drugs instead of applying FGF21 alone. The beneficial effect of the combined application may be because antidiabetic clinical medicines can reduce FGF21 levels, which may improve FGF21 sensitivity 132. Moreover, the performance of the antidiabetic drug exenatide is poor in T2DM patients with high baseline FGF21 levels, which was observed in a multicenter trial 133. In addition, FGF21 may be the downstream target of antidiabetic drugs, such as metformin 27, GLP-1 analog 133, and sodium/glucose cotransporter-2 inhibitors 25. In diabetic mouse models, researchers found that GLP-1 analogs can promote hepatocytes to produce FGF21, which inhibits hepatic glucose output 134. Moreover, GLP-1 analogs regulate WAT lipid metabolism in T2DM mice through the FGF21- liver kinase B1 (LKB1)- AMPK- ACC1 pathway 26.

The combined application of FGF21 and clinical anti-diabetic drugs has been reported in many preclinical studies. GLP-1 and FGF21 are the most common combinations. In diabetic mouse models, researchers designed a GLP-1 and FGF21 dual agonist with enhanced KLB binding affinity, which shows a superior glucose-lowering effect compared to GLP-1 or FGF21 alone 135. Another study also supported that sustained release of a GLP-1 and FGF21 dual agonist can protect diabetic mice from hyperglycemia 136. In addition, a drug delivery system used for the delivery of GLP-1 and FGF21 *in vitro* and *in vivo* was developed 137. Mesenchymal stem cells modified by FGF21 and GLP-1 transplantation are another way to combine GLP-1 and FGF21. Researchers transplanted mesenchymal stem cells into a T2DM mouse model and observed the therapeutic effects of T2DM 138. Apart from combining FGF21 and GLP-1, FGF21 synergizes with Forkhead box protein O1 inhibition, revealing the impact on normalizing glucose control in diabetic mice 139. In addition, studies have demonstrated that the combined application of FGF21 and insulin can increase the sensitization of FGF21 and insulin on metabolic effects in T2DM mice. Another study further investigated the effect of combined FGF21 and insulin on diabetic nephropathy and found better glucose and lipid amelioration effects than insulin or FGF21 alone 140.

In conclusion, many FGF21-based drugs, such as pegylated FGF21, FGF chimeras, and FGF21 gene therapy, have been designed to treat T2DM. Although the glucose-lowering effect of FGF21 is limited in humans, the combined application of FGF21 and clinical anti-diabetic drugs such as GLP-1 has shown a great glucose-lowering effect.

### 4.2 Obesity

FGF21 can be a biomarker that reflects the degree of obesity and the effectiveness of weight loss. Additionally, FGF21 can exhibit a weight loss effect by reducing energy intake and enhancing energy dissipation. FGF21-based drugs as well as FGF21 and GLP1 dual agonists, have been developed to treat obesity.

#### 4.2.1 Relationship between FGF21 and obesity

Obesity is a complex metabolic disease that can contribute to other metabolic diseases, such as T2DM and CVD 141. FGF21 has been considered a suitable biomarker for predicting many obesity-related situations. For example, recent studies have gradually focused on FGF21 levels in the pediatric population, not just adults. A cross-sectional and longitudinal analysis revealed that FGF21 was higher in obese hyperglycemic adolescents 142. Another study supported that FGF21 can be a suitable biomarker to predict the total intrahepatic lipid content and fatty liver in obese children 143. In addition, one study investigated the relationship between FGF21 and childhood obesity, and findings suggested that it is FGF21 deficiency instead of FGF21 resistance that results in insulin resistance in obese children 144. Moreover, FGF21 genetic variants might influence qualitative changes in nutritional behavior, such as food preferences, in children 145.

On the other hand, the biomarker function of FGF21 in weight loss has been discussed in many studies. One analysis selected a cohort of healthy overweight or obese subjects to complete a 12-month energy restriction pattern. The results indicated that FGF21 levels are not influenced by weight loss in a healthy obesity population 146. However, in obese adolescents, the level of FGF21 was increased after long-term interdisciplinary weight loss therapy, which can maintain the rest metabolic rate 147. Interestingly, in obese mice, weight loss can lead to decreased FGF21, which can be used to predict improved behavior after weight loss 148. Thus, the results on FGF21 levels post weight loss are not uniform. Consistently, one study that enrolled overweight and obese women found that changes in FGF21 levels differed among the subjects participating in the interdisciplinary intervention. Furthermore, they found that the adiponectin/leptin ratio was changed only in people with elevated FGF21 149. The changes in FGF21 levels after dietary interventions in different people can be used to assess the individual predisposition to weight gain over time. One study assessed FGF21 changes after 24 hours with different macronutrient contents and found that individuals with a blunted FGF21 response to a low-protein diet are at high risk for future weight gain 150.

Bariatric surgery is an essential weight-loss strategy that can effectively improve obesity and T2DM 151. Laparoscopic sleeve gastrectomy (LSG) is a typical operation of bariatric surgery. The FGF21 levels after LSG are not certain. One study containing 40 severely obese participants showed no effect of bariatric surgery, including LSG and Roux-en-Y gastric bypass surgery, on serum FGF21 152. However, in HFD-fed male Wistar rats, LSG can reduce weight with decreased FGF21 153. Consistently, one study reported that three months after ileal transposition metabolic surgery, serum FGF21 levels were lower in obese Zucker rats 154. Moreover, in research containing 137 obese patients with insulin resistance and high baseline FGF21, FGF21 concentrations were decreased after LSG-induced weight loss. They concluded that FGF21 concentrations appear to predict the insulin resistance change after weight loss 155. However, another study showed increased FGF21 after bariatric surgery in obese patients compared with a diet-induced weight loss strategy. That study suggested that the increased FGF21 is independent of insulin sensitivity, which shows the predicted effect of FGF21 as a marker of severe nutritional stress 156.

In conclusion, the FGF21 levels in obese subjects after weight loss therapy, such as dietary interventions and bariatric surgery, are changed. Although the study result about whether FGF21 levels are increased or decreased after interventions is not certain, the value of FGF21 to predict the predisposition to weight gain over time is worthy of being investigated.

#### 4.2.2 Mechanisms of FGF21-mediated anti-obesity effects

FGF21 can exhibit its weight loss effect by reducing energy intake and enhancing energy dissipation.

In obese minipigs, treatment with FGF21 leads to energy intake reduction and weight loss compared to the vehicle group 157. The reducing energy intake effect of FGF21 may contribute to its impact on food preference. It has been shown that serum FGF21 levels are associated with food and drug craving in humans 158. Although the effects of FGF21 on the preference for high-calorie foods are different by sex, FGF21 administration beneficially affects mice of both sexes 159. Mechanistically, one study demonstrated that mice cannot respond to protein restriction when the brain FGF21 coreceptor is deleted, which indicated that FGF21 can reflect protein status to the brain and make adaptive food choices 160. However, another *in vivo* study held that the effect of FGF21 administration on dietary protein restriction-induced weight loss is mediated by suppressing simple sugar consumption and promoting the intake of other macronutrients rather than altering protein preference 161. FGF21 can also increase appetite and stimulate food intake under a fasting state in zebrafish. The expression of FGF21 increases before meals and decreases after meals, which shows the effect of FGF21 on balancing energy and metabolism in different situations 162.

The brain receives FGF21 signaling and regulates food preference, while FGF21-induced energy expenditure mostly occurs in adipose tissues. FGF21-mediated thermogenesis in adipose tissue is the main mechanism by which FGF21 enhances energy expenditure. KLB is decreased in WAT and BAT during DM in mice, indicating the impaired FGF21 thermogenic effect during DM and obesity 163. In FGF21-deficient HFD mice, the expression of thermogenic-related genes such as Ucp1 is markedly reduced in BAT, leading to a reduction in the thermogenic response 164. Researchers have also investigated the thermogenic effect of FGF21 in primates. Whole transcriptome analysis in primate subcutaneous adipose tissues revealed corresponding gene network changes in lipid storage and metabolism after FGF21-induced weight loss 165. Mechanistically, beige fat thermogenesis was found to be related to the microRNA-182-5p-FGF21-acetylcholine pathway in rats. MicroRNA-182-5p is a crucial antiobesity molecule in adipocytes that promotes FGF21 expression and secretion in adipocytes. FGF21 stimulates acetylcholine synthesis and release from macrophages, which in turn activates the nicotine acetylcholine receptor of adipocytes, and thermogenic genes are consequently expressed 166. Notably, although most activation of BAT thermogenesis requires the help of Ucp1, endogenous FGF21 can prevent obesity by browning WAT in the absence of Ucp1 in mice 167. One recent *in vivo* study provided a different point of view that the Ucp1-independent pyrexic effect of FGF21 may not increase energy expenditure 168. Nonetheless, mice can still lose weight by decreasing food intake and increasing physical activity without thermogenic effects 169, suggesting that the FGF21-mediated balance of energy and metabolism is not just one-dimensional.

Apart from weight loss, FGF21 can improve obesity-induced metabolic disorders. Previous studies have found that exogenous FGF21 administration can exhibit great metabolic effects on HFD obese mice 170, and FGF21 deficiency will aggravate obesity-induced inflammation and inflammation-mediated atrophy in obese mouse skeletal muscle 171. Obesity-related inflammation is a critical element in the development of other metabolic diseases. One study found that FGF21 can efficiently ameliorate obesity-related inflammation in obese mice and 3 T3-L1 preadipocytes, which are mediated by the fibroblast growth factor receptor substrate 2-extracellular regulated protein kinases 1/2 signaling pathway 172.

#### 4.2.3 Preclinical and clinical trials of FGF21 in obesity

Many FGF21-based drugs have also been developed and studied in obese mice. For example, researchers utilized Lactococcus lactis NZ3900 to produce rhFGF21, which can activate BAT to reduce the bodyweight of *db/db* mice 173. Many studies have chosen to increase FGF21 expression through genetic plasmids. One study reported that injection with a liver-specific FGF21 expression nonviral plasmid in FGF21 knockout mice can restore FGF21 expression and regulate body mass 174. Another study utilized an adeno-associated viral vector to express FGF21 in mouse visceral adipose tissue, which resulted in reduced whole-body adiposity and adipose tissue macrophage inflammation 130. Moreover, researchers recently designed a novel FGF21 named LAPS-FGF21 with a longer half-life via conjugation to the Fc fragment of IgG4, which has shown an excellent therapeutic effect on obesity in mice 175. In addition, the FGF21 and GLP1 dual-agonist, which was developed by using a polypeptide linker to fuse GLP-1 and FGF21, also shows excellent potent weight-reducing effects in diabetic mice 136. Regarding clinical trials, in patients with obesity and T2DM, pegbelfermin (BMS-986036) has demonstrated its impact on improving high-density lipoprotein cholesterol and triglycerides in its randomized phase 2 study 126.

### 4.3 NAFLD/Nonalcoholic steatohepatitis (NASH)

FGF21 is a potential biomarker to reflect pathological processes of NAFLD, such as liver fat content and liver inflammation. The effect of FGF21 on protecting against NAFLD/NASH is targeted on the pathological characteristics of NAFLD/NASH, including reducing hepatocyte stress and hepatic steatosis as well as directly protecting against inflammation and fibrosis. Additionally, clinical trials of FGF21-based drugs, such as pegbelfermin, LLF580, and BIO89-100, have been performed on patients with NAFLD/NASH, and great effects have been observed.

#### 4.3.1 Relationship between FGF21 and NAFLD/NASH

NAFLD is more than 5% of hepatocytes with cytoplasmic triglyceride droplets and no evidence of other chronic liver conditions. NASH is the severe stage of NAFLD 176. The association between FGF21 and NAFLD has been confirmed among 3634 participants free of apparent CVD 177, and FGF21 is becoming a potential biomarker to reflect many pathological processes of NAFLD, such as liver fat content and liver inflammation. The FGF21 to adiponectin ratio has emerged as a biomarker to reflect the liver fat content of obese children 178. Serum FGF21 alone is also supported for use as a biomarker for NAFLD liver fat content in a randomized trial that enrolled 220 NAFLD patients with central obesity 179. Regarding the relationship between FGF21 and liver inflammation, researchers used a linear regression method to explore the impact of some liver inflammatory mediators on FGF21 and supported that FGF21 can be a biomarker for the liver inflammation process 180. NASH is the higher stage of NAFLD with severe liver inflammation, and another cross-sectional study demonstrated that FGF21 is associated with the severity of NASH 181. Apart from the potential to assist NAFLD/NASH diagnosis, FGF21 can help us predict NAFLD/NASH prognosis and treatment efficacy. Serum FGF21 levels can reflect the improvement of NAFLD in patients who received weight loss intervention in a prospective observational pilot study 182. FGF21 levels can also be used to evaluate the effects of liraglutide, a drug that can reduce liver fat content, in overweight patients with NAFLD 183.

#### 4.3.2 Mechanisms of FGF21 against NAFLD/NASH

FGF21 can protect against NAFLD/NASH by reducing hepatocyte stress and hepatic steatosis as well as directly protecting against inflammation and fibrosis, which is aimed at the pathological characteristics of NAFLD/NASH. For example, one *in vitro* study showed that dehydrovomifoliol-induced FGF21 can alleviate lipid accumulation in HepG2 cells 184. In mouse models, the overexpression of hepatocyte-specific FGF21 can ameliorate HFD-induced liver steatosis 185. Notably, the effect of FGF21 on liver steatosis is sex-specific in obese A(y) mice, showing more beneficial effects on males with melanocortin obesity 186. Another mouse study pointed out that fasting-induced FGF21 can activate autophagy-mediated lipid degradation in a Jumonji domain containing-3 (JMJD3)-dependent manner. Mechanistically, JMJD3 upregulates autophagy-network genes upon FGF21 signaling, resulting in hepatic autophagy and lipid degradation. Consistently, hepatic expression of JMJD3 is substantially decreased in NAFLD patients 187. In addition, one study suggested that hepatic six transmembrane protein of prostate 2 may be the downstream therapeutic target of FGF21 to improve NAFLD accompanying hepatic iron overload 188. They observed that rhFGF21 treatment ameliorates hepatic steatosis and reduces iron exporters by upregulating hepatic six transmembrane protein of prostate 2 expressions in oleic acid-treated HepG2 cells.

Regarding the effect of FGF21 on improving liver inflammation, FGF21 can regulate hepatic metabolic gene expression and hepatic mitochondrial function to improve inflammation. One study compared the treatment effect for NAFLD between FGF21 administration and caloric restriction in diet-induced obese mice. They performed a high-throughput quantitative polymerase chain reaction and found that FGF21 administration affects more hepatic genes than caloric restriction to improve steatosis and inflammation 189. Another study investigated the effects of the engineered FGF21 variant (LY2405319) in *ob/ob* mice with diet-induced NASH. The results showed that LY2405319 treatment reduces the symptoms of steatohepatitis and inflammatory markers by enhancing hepatic mitochondrial function 190. Moreover, FGF21 can also exert its anti-inflammatory effect by inhibiting the hepatocyte-Toll-like receptor 4-interleukin-17A pathway to prevent NASH from transitioning into hepatocellular carcinoma 191.

In addition, cardiovascular disease is the leading cause of death among NAFLD/NASH patients, and an imbalanced metabolic state is an important mechanism underlying NAFLD/NASH. Compared with other potential NAFLD/NASH drugs, apart from the effects on the pathological characteristics of NAFLD/NASH itself, FGF21 also delivers improvements in whole-body metabolism and cardiovascular risk profile, as mentioned above.

#### 4.3.3 Preclinical and clinical trials of FGF21 in NAFLD/NASH

There is no specific drug for NAFLD/NASH 192, and FGF21 has been a potential hot strategy for NAFLD/NASH. Drugs have been developed to treat NAFLD/NASH based on the beneficial pharmacological effects of FGF21. Efruxifermin is a long-acting FGF21 drug in clinical trials for NASH treatment. Researchers administered efruxifermin to Sprague Dawley rats weekly and found that it can reduce body weight without increasing sympathetic tone 193. The BALANCED program is a clinical study with NASH patients conducted at 27 centres in America. It is also a phase 2a trial for eftuxifermin. Eighty patients were divided into receive efruxifermin treatment (28/50/70 mg or placebo, subcutaneous injection, weekly, 16 weeks), which showed reduced hepatic fat fraction in patients with F1-3 stage NASH. Regarding the adverse event of FGF21 in clinical trials, the most common adverse event is grade 1-2 gastrointestinal events 194.

Pegbelfermin is a PEGylated FGF21 drug that has advanced to phase 2 clinical trials for NASH treatment. In phase 2a trials, pegbelfermin shows improvements in liver fat fraction reduction and decreases liver damage/fibrosis markers 126, 195. The FALCON program includes two phase 2b studies: subcutaneous injection with pegbelfermin to treat NASH, bridging fibrosis, and compensated cirrhosis 196. Patients in these two studies were divided into receiving pegbelfermin (10/20/40 mg) or placebo treatment every week for 48 weeks and then followed for an additional four weeks. This program will provide information such as dose selection to support the phase 3 program, which can better observe hard outcomes of FGF21, such as long-term survival or cirrhosis 197. Regarding safety issues, injection-site bruising and gastrointestinal conditions, such as diarrhea, are the most prevalent side effects, and most adverse events are mild 126. In addition, B1344 is a novel PEGylated FGF21 analog designed in recent years. Researchers administered B1344 to cynomolgus monkeys with NAFLD and methionine-deficient mice with induced NAFLD via subcutaneous injection for 11 weeks. These preclinical results indicated that B1344 administration can improve hepatic steatosis, inflammation, and fibrosis 198.

LLF580 is a novel FGF21 analog with an additional disulfide bond and a fused Fc domain. The clinical study of LLF580 comprised 61 obese participants divided into two groups and treated with 300 mg LLF580 or placebo via once-month subcutaneous injections for three months 199. LLF580 is beneficial in improving liver fat and injury, suggesting its potential to treat NAFLD. Notably, compared with other FGF21-based drugs administered once weekly, the administration frequency of LLF580 is once monthly, which provides additional patient convenience. However, this durable pharmacology was under a very high 300-mg single dose, and the cost-effective dose should be further investigated. In addition, GLP-1 and FGF21 dual agonists have also been studied in the treatment of NASH in recent years in HFD-induced obese mice 135.

### 4.4 Metabolic syndrome (MS)

MS is a series of risk symptoms for CVD and T2DM, including dyslipidemia, hypertension, hyperglycemia, and other metabolic disorders 200. The core of MS is insulin resistance. FGF21 has been identified as an insulin-dependent postprandial hormone 98 and a metabolic regulator that can improve lipid and glucose homeostasis 107. The levels of FGF21 in MS patients have been observed in many studies. Serum levels of FGF21 are positively associated with MS in patients with T2DM 201, and serum FGF21 levels in MS patients are higher than those in healthy volunteers after 12 weeks of supervised exercise 202. In addition, one American multiethnic longitudinal study investigated the role of FGF21 in accessing MS incidents, finding that people with higher FGF21 levels tend to develop MS 203.

Current studies show that FGF21 was a repressor of adipocyte lipolysis in both mice and humans, and treatment with FGF21 can reduce free fatty acids 204, 205. However, the glucose-lowering effects of FGF21 demonstrated in humans were attenuated compared with those in mice, which may account for the lack of BAT in humans compared with rodents. BAT is the target tissue of FGF21 that conducts acute insulin sensitization 206. Circulating lipid profile improvement caused by FGF21 does not accompany improvement in glycemic control, which reminds us to reshape the view of FGF21 as prevention instead of cure. For instance, FGF21 analogs can be used in obesity before T2D develops to improve FGF21 responsiveness and maintain insulin sensitivity in the longer term. FGF21 can be used with other current glycemic control therapies to compensate for its shortage and better exert its metabolic regulation effects.

### 4.5 Other metabolic diseases

Apart from the abovementioned metabolic disease, FGF21 is associated with other metabolic disorders or metabolic states. Plasma FGF21 is elevated in inherited metabolic diseases such as mitochondrial disorders in both mice and humans 207. In addition, in the acromegaly population, increased FGF21 levels are independently associated with the state of acromegaly 208. Moreover, plasma FGF21 can predict the development of metabolic comorbidities in patients with other disorders. For example, the serum FGF21 levels in acute Kawasaki disease patients are evaluated to predict the development of coronary artery lesions 209. FGF21 is used for psoriasis patients to predict the risk of cardiometabolic comorbidities 210. FGF21 may also monitor the metabolic abnormalities induced by antiretroviral drugs in HIV infection patients 211 and psychotropic drugs in bipolar mania patients 212.

## 5. FGF21 in CVMD: a mechanistic overview

### 5.1 Reduction of cardiometabolic risk factors

FGF21 is a novel metabolic regulator of energy homeostasis. It was first recognized because of reduced plasma glucose and triglycerides in obese mice 213. Subsequently, an increasing number of mechanisms by which FGF21 reduces cardiometabolic risk factors have been investigated. Table 1 shows the role of FGF21-based drugs in CVMDs. For metabolic disease, glucose and lipid profiles are fundamental factors in maintaining a healthy metabolism, which can be improved by FGF21 26, 130, 135, 138. Studies that investigated T2DM and FGF21 found that FGF21 can improve glucose homeostasis-related risk factors, such as glycated hemoglobin glycosylated hemoglobin A1c 142, insulin 97, and insulin resistance 108. For obese animals and patients, preclinical and clinical trials have indicated the effect of FGF21 on improving the serum lipid profile and reducing body weight 173-175. Regarding CVD, FGF21 can reduce the risk factors that influence the pathologic process of CVD, such as atherosclerosis 214, arterial stiffness 113, and blood pressure 86.

### 5.2 Molecular targets of FGF21

FGF21 improves CVMD by modulating multiple signaling pathways related to lipid and glucose metabolism (Table 2). The major molecular targets of FGF21 against CVMD are AMPK, SIRT1, autophagy-related molecules, and gut microbiota-related molecules.

An important molecular target of FGF21 involved in the cardiometabolic protective effects is AMPK, a decisive energy metabolism regulator 215. AMPK-mediated signaling pathways can regulate many cell activities related to cardiometabolic processes. In endothelial cells, FGF21 can mediate the FGF21-calcium/calmodulin-dependent protein kinase 2-AMPKα pathway to exert antioxidative effects 114. Moreover, the FGF21-LKB1-AMPK-ACC1 pathway can prohibit the synthesis of triglycerides in macrophages, which further prohibits foam cell formation 26. AMPK-related antioxidative effects and lipid-lowering effects can also be observed in cardiomyocytes via the FGF21-AMPK-Akt2-Nrf2 pathway and FGF21-AMPK-ACC pathway 110, respectively. The AMPK-mediated cell activities mentioned above can inhibit the pathological process of atherosclerosis and consequently CVD. In addition, AMPK-mediated signaling pathways also influence the pathological process of metabolic disease. For example, in mice, the FGF21-AMPK-ACC pathway can increase beta cell survival and insulin secretion 97, and the FGF21-AMPK-mTOR pathway 100 can increase islet autophagy. Increased beta cells and improved beta cell function can regulate the serum glucose level and the process of T2DM.

Apart from the effect on AMPK, one *in vitro* study indicated that FGF21 can target SIRT to inhibit inflammatory oxidative stress in CVMD 216. Inflammatory oxidative stress and related cell death are essential in the development of atherosclerosis. The FGF21-SIRT1 pathway can prohibit reactive oxygen species accumulation in cardiomyocytes, reducing apoptosis 48. Consistently, FGF21 can reduce endothelial senescence by targeting SIRT1 83.

Under nutrient deprivation, autophagy is essential for energy homeostasis and cellular survival, and studies on FGF21 mediating autophagy are increasing. FGF21 mediates autophagy-induced cholesterol efflux in macrophages to inhibit macrophages from becoming foam cells by increasing activated kinase C receptor 1 in apoE^-/-^ mice 54. FGF21-induced autophagy can also alleviate H/R injury and cardiac steatosis in cardiomyocytes 73. In addition, islet autophagy and hepatic autophagy are essential to maintaining glucose and lipid homeostasis. FGF21 can mediate islet autophagy via the FGF21-AMPK-mTOR pathway in mice 100, and the effect of FGF21 on hepatic steatosis and insulin resistance in mice occurs via the FGF21-JMJD3 histone demethylase pathway 187.

The gut microbiota is another target by which FGF21 can reshape host inflammation and metabolic health. Many studies have reported that gut microbiota alterations are related to FGF21 levels. In a randomized controlled-feeding trial that enrolled 117 overweight adults, the results showed that fried meat consumption induced gut microbiota and metabolism changes that were associated with changes in FGF21 217. Similar results were also observed in gut microbiota transplantation trials. One study transplanted the gut microbiota into mice and found that the increase in gut butyrate is associated with elevated FGF21 in transplanted mice 218. Another study used microbiota transplantation to treat human recurrent clostridioides difficile infection and observed gut microbiota and bile acid profile restoration associated with an upregulated bile acid-farnesoid X receptor-FGF pathway 219. The FGF21-β-hydroxybutyrate-NLRP3 axis is another pathway in the gut microbiota. FGF21 can stimulate ketogenesis in the liver. Hepatic β-oxidation of fatty acids produces β-hydroxybutyrate, which further inhibits NLRP3 and modulates gut inflammation 220.

## 6. Summary of FGF21 in CVMD

### 6.1 FGF21: as a biomarker for metabolic and cardiovascular diseases

Although the FGF21 test is not a routine and standard test in hospital laboratories, current studies have observed that FGF21 is upregulated in metabolic and other disorders and realized its potential value as a diagnostic and prognostic biomarker for metabolic diseases. As mentioned earlier, FGF21 is considered a biomarker for predicting the presence of T2DM. In addition, FGF21 has the potential to assess the severity of the disease and predict long-term prognosis. For example, a lower level of FGF21 predicts a better prognosis in DM patients. FGF21 levels correlate with the severity of steatohepatitis, which can help identify patients with NASH at risk of disease progression 181. Compared to the traditional biomarker used in clinical laboratories, FGF21 still showed potential. One study indicated that FGF21 seems superior to other adipokines and can be recommended as an alternative to the glucose tolerance test 93.

The role of FGF21 as a biomarker in CVD is controversial. In some clinical studies, FGF21 is considered a biomarker for predicting the presence of atherosclerosis and cardiovascular events 41-47. One study also showed that the identification effect of FGF21, which identifies left ventricular systolic function and cardiac death, was not inferior to serum N-terminal pro-brain natriuretic peptide in patients 221. In addition, mechanistic studies revealed that FGF21 can act directly on the cardiovascular system and may be used as an early biomarker of CVD. FGF21 can induce cardiac protection by preventing cardiac lipotoxicity, oxidative stress, and inflammation by directly binding to the FGFR of the heart 222. However, in another multiethnic clinical study, FGF21 was not a CVD biomarker in atherosclerosis patients without known CVD 44. Previous studies also showed that cardiac protection may contribute to the beneficial effects of FGF21 as a metabolic regulator, which can regulate systemic glucose-lipid metabolism disturbance, the key risk factor for CVD 3. Moreover, although FGF21 can be produced by the heart and act on the heart in an autocrine way, circulating levels of FGF21 are now known to be derived primarily from the liver 13. Therefore, the circulating levels of FGF21 should not directly reflect the CVD-related pathophysiological process and should be used as a biomarker of CVD.

In summary, FGF21 is a valuable biomarker for metabolic diseases and deserves further study to translate the biomarker potential into clinical laboratories. Regarding the role of FGF21 as a biomarker in CVD, more studies are needed to investigate the relationship between the effect of FGF21 on the systemic and the heart, and more careful thoughts are needed on the meaning of circulating liver-derived FGF21 as a biomarker in CVD.

### 6.2 Preclinical and clinical trials of FGF21 in CVMD

Preclinical studies about the role of FGF21 in CVMD are summarized in Table 3. As mentioned before, FGF21 can improve the development of CVMDs, including atherosclerosis, CAD, MI, H/R injury, HF, T2DM, obesity, and NASH, *in vivo* and *in vitro*. Moreover, clinical trials of FGF21-based drugs are mainly performed on patients with NAFLD/NASH and related hypertriglyceridaemia, which are summarized in Table 4. In addition to pegbelfermin 196 and LLF580 199, BIO89-100 is another novel FGF21-based drug that was presented recently. The impact of BIO89-100 on reducing liver fat and lowering triglycerides is still under further investigation.

## 7. Conclusions and outlook

In the past decade, a great stride has been made in our understanding of the pivotal role of FGF21 in CVMD, the leading cause of death worldwide. Regardless of the molecular mechanisms or clinical applications, there are many contradictions regarding FGF21, which increases the difficulty of successful clinical application. First, the pharmacological effects of FGF21 are species specific. The most significant difference is that the glucose-lowering effects of FGF21 analogs in humans are attenuated compared with those in mice 126, 129, which may account for the lack of BAT in humans compared with rodents 206. Another controversial impact of FGF21 is bone loss. Mouse studies have suggested that FGF21 promotes bone loss, but this effect in humans is conflicting 223, 224. Second, the pharmacologic and physiological effects of FGF21 are different under different stimuli, tissue sources, and experimental models. For example, both fasting and feeding can induce the production of FGF21 9. FGF21 can decrease the preference for high-calorie foods 159 as well as increase appetite 162. Moreover, FGF21 is significantly elevated in obesity and DM, which may be attributed to 'FGF21 resistance' 225. However, obese rodents and humans in this potentially FGF21-resistant state can still respond to FGF21 at pharmacological levels 226. Contradictions on FGF21 suggest that the metabolic balance mediated by FGF21 is dynamic and involves multiorgan crosstalk instead of one-dimensional crosstalk 227. The nervous system plays an essential role in coordinating complex and dynamic FGF21-mediated responses. Identification of the neural circuits related to FGF21 may help to better understand the nuanced regulatory effects of FGF21 and will be an important future direction 228. Further investigations related to this 'contradictory molecule' are needed to promote the application of FGF21.

Regarding the clinical application of FGF21, the focus of FGF21 clinical trials is on NAFLD/NASH based on its powerful lipid profile benefits, which have been processed in phase 2b studies 196. However, a study on FGF21 clinical application did not meet the primary endpoints of glycemic control 126, suggesting that further studies are warranted to utilize FGF21 as a stand-alone medication for T2DM. Actually, FGF21 can act as a 'sensitizer' instead of a 'regulator' in CVMD. Combining FGF21 and clinical antidiabetic drugs is a potent therapeutic in T2DM 132. In addition, FGF21 administration methods are continuously being developed to overcome the poor pharmacokinetics of natural FGF21, such as FGF21 gene therapy and tissue target therapy 130. In addition to FGF21 analogs, FGF21-based drugs, such as FGF21 receptor agonists, FGF21-degrading protease inhibitors, and FGF21 transcription factor activators, have also been investigated (Figure 3B) 229. Notably, beyond the clinical application of FGF21-based drugs, FGF21 has great potential as a biomarker for metabolic disease diagnosis and prognosis.

In conclusion, the performance of FGF21 between pharmacology and physiology as well as among different species is obviously different. Considering the complexity and heterogeneity of FGF21, personalized FGF21-based and combination pharmacotherapies used with other current metabolic therapies should be given more attention in the future.

## Figures and Tables

**Table 1:** Overview of FGF21-based drugs in cardiometabolic disease

FGF21-based drugs	Target	Effect	Species	Reference
**Agents that increase FGF21 expression**				
The renal sodium/glucose cotransporter-2 inhibitors (Canagliflozin)	AMP-activated protein kinase (AMPK) ↑- FGF21 ↑	Fat mass reduction and lipolysis activation	Mouse	25
Glucagon-like peptide 1 analogue (Liraglutide)	FGF21↑	Triglycerides synthesis ↓	Mouse	26
Metformin	Activating transcription factor 4 (ATF4) ↑-FGF21 ↑	Anti-obesity and anti-diabetes effects	Mouse and human	27
Intestinal serine protease inhibition (Camostat)	FGF21 ↑	Food intake and body weight ↓	Mouse	230
Peroxisome proliferator-activated receptor alpha (PPARα) modulator (Pemafibrate/K-877)	PPARα ↑-FGF21 ↑	Management of atherogenic dyslipidaemia	Type 2 diabetes (T2DM) patients	21
Class I-specific histone deacetylase inhibitor (MS-275)	cAMP-responsive element-binding protein H (CREBH) ↑-FGF21 ↑	Lipid metabolism-related gene expressions ↑	Mouse	22
Dietary Cyanidin-3-Glucoside	FGF21 ↑	Body weight and impairment of glucose tolerance ↓	Mouse	231
Dichloroacetate	AMPK ↑- FGF21 ↑	Against atherosclerosis	Mouse	232
Ishige okamurae extract	FGF21 ↑	Hyperglycemia and Body weight ↓	Mouse	233
Mediterranean tomato-based sofrito sauce	FGF21 ↑	Healthy effects on glucose metabolism	Rat	234
Carbon monoxide	ATF4 ↑-FGF21 ↑	Anti-inflammatory, antiproliferative, and antiapoptotic effects	Mouse	23
Berberine	AMPK ↑-FGF21↑	The beneficial metabolic effect	Primary mouse hepatocytes	235
Berberine	Sirtuin1 (SIRT1) ↑-FGF21↑	Hepatic lipid metabolism and energy expenditure ↑	Mouse	236
Neohesperidin	FGF21↑	Lipid-regulating effects	Mouse	20
Diet polyphenol curcumin	FGF21↑	Metabolic beneficial effects	Mouse	237
A combined docosahexaenoic acid-thyroid hormone protocol	FGF21↑	Energy expenditure ↑	Rat	238
The TAZ activator 2-butyl-5-methyl-6-(pyridine-3-yl)-3-[2'-(1H-tetrazole-5-yl)-biphenyl-4-ylmethyl]-3H-imidazo[4,5-b] pyridine] (TM-25659)	ATF4 ↑-FGF21↑	Insulin resistance and inflammation ↑	Mouse	239
CCR4-NOT Transcription Complex Subunit 6 Like (CNOT6L) inhibitor	CNOT6L↓-FGF21↑	Treat metabolic syndrome	Mouse	24
Haem-regulated eukaryotic translation initiation factor 2α kinase (HRI) activators	FGF21↑	Treat T2DM	Mouse	240
Lentivirus-Mediated human FGF21	FGF21↑	Treat T2DM	Mouse	131
**FGF21-degrading protease inhibitors**				
Fibroblast activation protein (FAP) inhibition	FGF21↑	Treat obesity-related metabolic disorders	Cynomolgus monkeys	28
**Engineered FGF21 analogues**				
Pegbelfermin	Binding to FGFR1 and β-Klotho	Improved metabolic parameters	Obesity and T2DM patients	126
Efruxifermin	Binding to FGFR1 and β-Klotho	Hepatic fat fraction ↓	NASH patients	194
LLF580	Binding to FGFR1 and β-Klotho	Triglycerides and hepatic fat ↓	Obesity patients	199
BIO89-100	Binding to FGFR1 and β-Klotho	Improve serum triglycerides	Hypertriglyceridemia patients	241
**FGF21-receptor agonists**				
C3201	Binding to FGFR1 and β-Klotho	Treat obesity	Monkeys	242
The agonistic anti-FGFR1 antibody (R1MAb1)	Binding to FGFR1	Treat obesity and diabetes	Mouse	243
FGF21-mimetic monoclonal antibodies (mimAb1)	Binding to β-Klotho	Treat obesity	Cynomolgus monkeys	244
GLP-1 and FGF21 dual agonist	Binding to β-Klotho	Treat diabetes	Mouse	135

**Table 2:** Molecular targets of FGF21 in different cell types in CVMD

Cell type	Molecular targets	Observed effects	References
Endothelial cells	FGF21-**SIRT1**	Endothelial senescence↓	48
	FGF21-**TET2**-UQCRC1-ROS	Ox-LDL-induced HUVEC pyroptosis ↓	49
	FGF21-**Fas**	HUVEC apoptosis ↓	50
	FGF21-**NFkappaB**	Atheromatous plaques ↓	52
	FGF21-**CaMKK2**-AMPKα	Antioxidative; dilate the aorta	114
Macrophage	FGF21-**NFkappaB;** FGF21-**RACK1**-autophagy; FGF21-**LKB1-AMPK**-ACC1	Foam cells formation and macrophage migration ↓; synthesis of TG ↓	53,54,26
Smooth muscle cells	FGF21-**Syk**-NLRP3 inflammasome	Proliferation and migration ↓	58
Cardiomyocytes	FGF21-**SIRT1**-LKB1; FoxO1; FGF21-**Ucp2/3; Sod2**	ROS accumulation and cardiomyocyte apoptosis ↓	83,84
	FGF21-**microRNA-143**-EGR1-Na_V_1.5/Kir2.1; FGF21-**Na_V_1.5/Kir2.1**	Ventricular arrhythmias in post-infarcted hearts ↓	70,71
	FGF21-**AMPK**-Akt2-Nrf2; FGF21-**AMPK**-ACC	Antioxidative effect; lipid-lowering effect	110
	FGF21-**PI3K**-Akt; FGF21-**ALCAT1**	Oxidative stress and apoptosis ↓	68
	FGF21-**EGR1**; FGF21-**galectin-3**	Collagen synthesis and inflammation after myocardial infarction ↓; pathological cardiac remodeling ↓	67,74
	FGF21-**Beclin1/Vps34**-autophagy; FGF21-**microRNA-145/ Angpt2/autophagy**	Hypoxia/reoxygenation injury ↓	73,72,75
	FGF21-**autophagy**	Cardiac steatosis ↓	82
Cardiac fibroblasts	FGF21-**TGF-β1**-Smad2/3-MMP2/9	Cell apoptosis and collagen production ↓	69
Beta cells	FGF21-**ERK1/2;** FGF21-**Akt;** FGF21-**PI3K**-Akt; FGF21-**AMPK**-ACC; FGF21-**PPARδ/γ**	Beta-cell survival and insulin secretion ↑	40,97,99
	FGF21-**AMPK**-mTOR	Islet autophagy ↑	100
Adipocytes	FGF21-**ERK1/2**-CCL11; FGF21-**acetylcholine**-acetylcholine receptor	Beiging of white adipose tissue ↑; anti-inflammatory effects ↑	38,166,172
	FGF21-**adiponectin**	Neointima formation and macrophage inflammation ↓; insulin resistance ↓	60,76,104,105
	FGF21-**NF-kappaB**-TNF-α	Inflammation ↓; glucose uptake ↑	108
Hepatocyte	FGF21-**hepatic sterol regulatory element-binding protein-2**	Cholesterol synthesis ↓	60
	FGF21-**mTORC1**	Hepatic insulin resistance ↓; glycogen synthesis ↑; liver steatosis ↓	103,185
	FGF21-**JMJD3 histone demethylase**	Hepatic autophagy and lipid degradation ↑	187
	FGF21-**hepatic six transmembrane protein of prostate 2**	Hepatic steatosis and insulin resistance ↓	188
	FGF21-**TLR4**-IL-17A	Prevent nonalcoholic steatohepatitis- hepatocellular carcinoma transition	191

**Abbreviations:** Fibroblast growth factor 21 (FGF21), silent information regulator 1 (SIRT1), tet methylcytosine dioxygenase (TET2), ubiquinol cytochrome c reductase core protein I (UQCRC1), reactive oxygen species (ROS), low density lipoprotein (LDL), human umbilical vein endothelial cell (HUVEC), nuclear factor kappa light chain enhancer of activated B cells (NF-κappaB), calcium/calmodulin-dependent protein kinase kinase 2 (CaMKK2, also known as CaMKKβ), adenosine 5'-monophosphate -activated protein kinase (AMPK), Activated kinase C receptor 1 (RACK1), liver kinase B1 (LKB1), acetyl coenzyme A carboxylase (ACC), triglyceride (TG), spleen tyrosine kinase (Syk), NOD-, LRR- and pyrin domain-containing 3 (NLRP3), forkhead box protein O1 (FoxO1), mitochondrial uncoupling proteins (Ucp2 and Ucp3), MnSOD (Sod2), early growth response protein 1 (EGR1), voltage-gated sodium channels 1.5 (Na_V_1.5), potassium inwardly-rectifying channels 2.1 (Kir2.1), nuclear transcription factor-E2-related factor 2 (Nrf2), phosphatidylinositol 3-kinase (PI3K), lysocardiolipinacyltransferase-1 (ALCAT1), vacuolar protein sorting 34 (Vps34), angiopoietin-2 (Angpt2), transforming growth factor-β1 (TGF-β1), drosophila mothers against decapentaplegic protein (Smad), matrix metalloproteinase (MMP), extracellular regulated protein kinases (ERK), peroxisome proliferators-activated receptors (PPAR), C-C Motif Chemokine Ligand 11 (CCL11), tumour necrosis factor-α (TNFα), mammalian target of rapamycin (mTOR), jumonji domain containing-3 (JMJD3), toll-like receptor 4 (TLR4), interleukin-17A (IL-17A).

**Table 3:** Role of FGF21 in cardiometabolic disease in preclinical studies

Animal models	Recombinant human FGF21	Observed effects	References
apoE^-/-^ mice	1mg/kg/d, *s.c.*, 14wk; 10mg/kg/d, *s.c.*, 12wk; 0.1 mg/kg, *i.p.*, 5wk	Anti-atherosclerotic effect	51,54,61
HFD induced- atherosclerosis mice	6mg/kg/d,* i.v.*, 40d	Anti-atherosclerotic effect	52
APOE*3-Leiden.CETP mice	1mg/kg/times, 3 times/w, *s.c.*, 16wk	Anti-atherosclerotic effect	62
SIRT1-iKO mice	2.5mg/kg/d, *i.p.*, 4wk	Ang II-induced cardiac hypertrophy ↓	83
MI rats; FGF21 KO MI rats	7μg/kg/d, *i.p.*, 4wk; 1×10^9^ pfu/mouse, *i.m.*; 1 μg/kg, 5 μg/kg, and 10 μg/kg, *i.v.*	Postinfarcted inflammation; fibrosis; arrhythmias ↓	67,76,71
Wistar rat myocardial I/R injury model	0.1 mg/kg/d, *i.p.*, 4wk	Myocardial ischemia-reperfusion injury ↓	72
FGF21-KO mice	0.1 mg/kg/d, osmotic pump, 4wk	Restores subcutaneous adipose tissue mass and reverses insulin resistance	104
Obesity mice	1 mg/kg/d, *i.p.*, 4wk	Insulin resistance ↓	105
Streptozotocin-treated mice	5mg/kg, twice/w, *s.c.*	Normalizes blood glucose without improving β-cell function	101
	10mg/kg, twice/w, *i.p.*, 4wk	Improve diabetic retinopathy	122
*Db/db* mice	1 mg/kg/d, *i.p.*, 5wk; FGF21-FGF19 chimera: 0.6 mg/kg/d, *s.c.*, 7d	Improving hyperglycemic and alleviating inflammation	108,129
	1.5mg/kg, twice/d, *s.c.*, 2wk	Improves neurological outcomes following focal ischemic stroke	117
	1mg/kg/w, *s.c.*, 2wk	Therapeutic potential for diabetes and NASH	135
T2DM rats	Injected the livers with lentivirus containing hFGF21 (50-μL volume with a titer of 5×10^9^ viral particles/ml)	Liver lipid droplet content ↓; insulin sensitivity ↑; glycogen synthesis ↑	131
Insulin-resistant BTBR mice	FGF21 gene therapy targeted visceral adipose tissue	Insulin sensitivity ↑; glycemic processing ↑; body weight ↓; hepatic steatosis ↓	130
T2DM mouse model	FGF21 and GLP1 modified mesenchymal stem cells transplantation	Lipid metabolism ↑; blood glucose ↓	138
T2DM cynomolgus monkeys	1mg/kg/d, *s.c.*, 4wk	Blood glucose ↓	128
Obese Göttingen minipigs	0.1 mg/kg/d for 9.5 weeks and 0.3 mg/kg/d for 4.5 weeks, *s.c.*, 14wk	Food intake ↓; body weight ↓	157
*Db/db* mice	Recombinant Lactococcus lactis NZ3900 expressing bioactive human FGF21: 200 μl/mice/d, *p.o.*, 13wk	Body weight ↓	173
*Db/db* mice; *ob/ob* mice	GLP-1 and FGF21 dual agonist: 1000nmol/kg; 0.5-1mg/kg	Body weight ↓; blood glucose ↓; NAFLD activity score ↓	136,135
Diet-induced obesity mouse	Long-acting FGF21: 75nmol/kg	Body weight ↓; blood glucose ↓	175
	0.6mg/kg/d, *s.c.*, 18d	Improve liver steatosis and inflammation	189
*Leptin-deficient ob/ob* mice	5mg/kg, *i.p.*, 3wk	Prevent NASH	190
FGF21-KO mice	Unknown dose, *s.c.*, 4wk	Liver steatosis ↓; peroxidative damage ↓	30
Cynomolgus monkeys with NAFLD	0.5-2mg/kg, 1-2times/w, *s.c.*, 11wk	Hepatic steatosis, inflammation, and fibrosis ↓	198
Obesity, T2DM, HF, and renal failure mouse models	Gene therapies based on 3 longevity associated genes included FGF21	Improve all 4 diseases	87

**Abbreviations:** subcutaneous injection (*s.c.*); intravenous injection (*i.v.*); intraperitoneal injection (*i.p.*); intramuscular injection (*i.m.*); per os (*p.o.*); apolipoprotein E^-/-^ (apoE^-/-^); high-fat diet (HFD); silent information regulator 1 (SIRT1); myocardial infarction (MI); ischemia/reperfusion (I/R); Angiotensin II (Ang II); nonalcoholic fatty liver disease (NAFLD); nonalcoholic steatohepatitis (NASH); type 2 diabetes (T2DM); heart failure (HF).

**Table 4:** Clinical trials involving FGF21

FGF21	Condition or disease	Clinical trial ID	Outcome	References
Pegbelfermin	Obesity and T2DM	NCT02097277	HbA1c -; high-density lipoprotein ↓; triglyceride ↓; adiponectin ↑	126
Pegbelfermin	Obesity and NAFLD	NCT02413372	Hepatic fat fraction↓	195
Pegbelfermin	FALCON 1: NASH with stage 3 liver fibrosis; FALCON 2: NASH with compensated cirrhosis	FALCON 1: NCT03486899; FALCON 2: NCT03486912	Improvement in fibrosis	196
Efruxifermin	NASH	NCT03976401	Hepatic fat fraction↓	194
LLF580	Obese adults with modest hypertriglyceridemia	NCT03466203	Triglycerides and hepatic fat↓	199
BIO89-100	Severe hypertriglyceridemia	NCT04541186	Improvement in serum triglycerides	241

**Abbreviations:** nonalcoholic fatty liver disease (NAFLD); nonalcoholic steatohepatitis (NASH); type 2 diabetes (T2DM); glycosylated haemoglobin A1c (HbA1c).

## Abbreviations

CVMD: cardiovascular and metabolic disease
FGF21: fibroblast growth factor 21
CVD: cardiovascular diseases
MI: myocardial infarction
HF: heart failure
DM: diabetes mellitus
MS: metabolic syndrome
FGF: fibroblast growth factor
FGFR: FGF receptor
KLB: β-Klotho
BAT: brown adipose tissue
PPAR: peroxisome proliferators-activated receptors
BBB: blood-brain barrier
WAT: white adipose tissue
Ucp1: uncoupling protein 1
CAD: coronary artery disease
T2DM: type 2 diabetes mellitus
NAFLD: nonalcoholic fatty liver disease
apoE^-/-^: apolipoprotein E^-/-^
rhFGF21: recombinant human FGF21
HUVECs: human umbilical vein endothelial cells
SIRT1: silent information regulator 1
NLRP3: NOD-, LRR- and pyrin domain-containing 3
NF-κappaB: the nuclear factor kappa light chain enhancer of the activated B-cell
AMPK: adenosine 5'-monophosphate-activated protein kinase
HFD: high-fat diet
AP: angina pectoris
PI3K: phosphatidylinositol 3-kinase
Akt: protein kinase B
I/R: Ischemia/reperfusion
HFrEF: HF with reduced ejection fraction
HFpEF: HF with preserved ejection fraction
ACC: Acetyl coenzyme A carboxylase
mTOR: mammalian target of rapamycin
Nrf2: nuclear transcription factor-E2-related factor 2
GLP-1: glucagon-like peptide-1
LKB1: liver kinase B1
LSG: Laparoscopic sleeve gastrectomy
JMJD3: Jumonji domain containing-3
MS: Metabolic syndrome
